# A systematic review of the effectiveness of digital interventions for illicit substance misuse harm reduction in third-level students

**DOI:** 10.1186/s12889-019-7583-6

**Published:** 2019-09-09

**Authors:** Samantha Dick, Eadaoin Whelan, Martin P. Davoren, Samantha Dockray, Ciara Heavin, Conor Linehan, Michael Byrne

**Affiliations:** 1 School of Public Health, University College Cork, Cork, Ireland; 2 School of Applied Psychology, University College Cork, Cork, Ireland; 3 Sexual Health Centre, Cork, Ireland; 4 Health Information Systems Research Centre, Cork University Business School, University College Cork, Cork, Ireland; 5 Student Health Department, University College Cork, Cork, Ireland

**Keywords:** Mhealth, Harm reduction, Student, Substance misuse

## Abstract

**Background:**

Illicit substance misuse is a growing public health problem, with misuse peaking among 18–25 year-olds, and attendance at third-level education identified as a risk factor. Illicit substance misuse has the potential to harm mental and physical health, social relationships, and impact on academic achievements and future career prospects. Digital interventions have been identified as a vehicle for reaching large student populations and circumventing the limited capacity of student health services for delivering face-to-face interventions. Digital interventions have been developed in the area of alcohol and tobacco harm reduction, reporting some effectiveness, but the evidence for the effectiveness of digital interventions targeting illicit substance misuse is lacking. This review aims to systematically identify and critically appraise studies examining the effectiveness of digital interventions for illicit substance misuse harm reduction in third-level students.

**Methods:**

We systematically searched ten databases in April 2018 using keywords and database specific terms under the pillars of “mHealth,” “substance misuse,” and “student.” To be eligible for inclusion, papers had to present a measure of illicit substance misuse harm reduction. Included articles were critically appraised and included in the qualitative synthesis regardless of quality.

**Results:**

A total of eight studies were included in the qualitative synthesis. Studies reported harm reduction in terms of substance misuse or initiation, as consequences or problems associated with substance misuse, or as correction of perceived social norms. Overall, five out of the eight studies reported at least one positive outcome for harm reduction. The critical appraisal indicated that the study quality was generally weak, predominantly due to a lack of blinding of study participants, and the use of self-reported substance misuse measures. However, results suggest that digital interventions may produce a modest reduction in harm from illicit substance misuse.

**Conclusions:**

The results of this review are positive, and support the need for further high-quality research in this area, particularly given the success of digital interventions for alcohol and tobacco harm reduction. However, very few studies focused solely on illicit substances, and those that did targeted only marijuana. This suggests the need for further research on the effectiveness of this type of intervention for other illicit substances.

**Trial registration:**

This review is registered on PROSPERO, ID number: CRD42018097203.

## Background

Illicit substance misuse in third-level student populations is an emerging public health issue, with annual prevalence growing gradually from 34% in 2006, to 43% in 2018 [1]. Rates of current illicit substance misuse are highest among 18–25 year-olds, with 23% reporting current misuse in 2016 [2, 3]. A significant proportion of this age group will spend a number of years in third-level education, in colleges and universities [4], providing a unique opportunity to deliver harm reduction interventions. Despite a recent decrease in marijuana misuse [1], 18% of third-level students report annual misuse of illicit substances other than marijuana [1], and just under a quarter of third-level students report current misuse of any illicit substance [1, 4–6]. Illicit substance misuse in third-level student populations is an under-researched area [7], as much of the research focuses on second-level populations [4]. Of the available research, the majority is United States (US) and European based, with considerable variation in prevalence rates. For example, the National Student Drug Survey in Ireland reported a lifetime prevalence of 82% among Irish students [8].

The transition from second-level to third-level education involves a complex process of “finding your place,” negotiating the old life left behind and the new life ahead [9]. As students try to fit into their new life, new relationships with college friends quickly begin to form the principal source of social support [9, 10]. Many students move away from the family home and experience freedom from parental supervision for the first time, which may lead to opportunities for illicit substance misuse to occur [11]. Students who live away from their parents report a four-times higher illicit substance misuse prevalence than students who live at home with their parents [7]. What may initially be regarded as a period of experimentation can sometimes lead to the formation of new habits, some of which may have an adverse effect on the student’s wellbeing [12]. Adjusting to a new environment, along with balancing an increased workload, can present students with stresses that exceed their resources to cope. This transitional period may heighten stress for students who may be ill-equipped to cope with the academic and social pressures they are met with [13, 14]. The temporary pleasure-inducing properties of illicit substances, in the short-term, can appear attractive to students, particularly as a means to cope [15, 16] as well as for recreational purposes, as part of the perceived normative experiences in college, and perceived peer influences, for enjoyment and as part of social experiences [17]. Students have also reported using illicit substances for management of their emotions [18]. For example, illicit substance misuse is particularly high during exam periods when students experience an increase in stress levels [19].

Research comparing third-level students with their non-student peers has generally found that substance misuse is lower in third-level student populations [1, 20]. However there are some notable differences; in particular, third-level students have the highest rate of amphetamine misuse, likely as a means to improve academic performance [1]; and have been found to be four-times more likely than non-students to have taken ketamine in the last 12 months [7]. Additionally, The United Nations Office on Drugs and Crime identifies attendance at third-level education a risk factor for cannabis misuse [21], and first-year students are at an increased risk of substance misuse [20]. Misuse of illicit substances is defined by the World Health Organisation as “use of a substance for a purpose not consistent with legal or medical guidelines” [22], and further by the National Institute on Drug Abuse as “the repeated use of a substance to produce pleasure, alleviate stress, and/or alter or avoid reality” [23]. Misuse of illicit substances can have far-reaching consequences for students, particularly if misuse begins early in the college career [24] by negatively impacting academic outcomes [20, 25], increasing drop-out rates [24, 26–28], delaying graduation [24], leading to expulsion or suspension [29], or failure to attain a degree [30], and potentially impacting career trajectories [31]. Students have also reported missing classes [32] and receiving a lower grade [29] as a result of their illicit substance misuse.

Outside of academic achievement, misuse of illicit substances can result in many other immediate and long-term, personal and societal harms including; engaging in risky sexual behaviours [20], being aggressive or engaging in violent behaviour [4, 20, 29], causing panic attacks, insomnia and nausea [4, 32]. Other short-term harms can be more severe including; seizures, memory loss, and unconsciousness [4]. Longer term effects include weight loss, teeth problems, and impact on sleep quality [33]. Illicit substance misuse can also have a profound impact on mental health [20] and has been linked to increased risk of depressive symptoms, deliberate self-harm, suicidal ideation, and suicidal attempts [34]. Harms from misuse are not limited to physical and mental effects. They can have a damaging impact on relationships and occupations [3, 26, 29], negatively affect financial situations [35], and lead to legal problems [20, 29], with a criminal record limiting future travel and career prospects [35]. In a study by Palmer et al. [29], almost half of respondents reported minor harms, such as embarrassment or guilt, whereas 17–19% reported more significant harms, such as failing to fulfil role function, or losing interest in activities. Similarly, Bennet and Holloway [4] reported that 16% of participants reported significant harms such as psychosis, loss of mobility, and unconsciousness.

Harm from substance misuse in third-level students is a pertinent public health issue, and although many interventions have been designed to target alcohol and tobacco use [36–41], much fewer have focused specifically on illicit substance misuse. Of the interventions that do target illicit substances, the predominant focus is on marijuana misuse. Third-level institutions are ideally placed to intervene and reduce the harm from illicit substance misuse, but limited resources and increasing demand have also meant that access to face-to-face healthcare interventions is constrained, with student health services struggling to meet the demands of large student populations [42]. Online and mobile platforms, such as websites and mobile phone applications have been lauded as an effective platform for delivering interventions due to their relatively low-cost and always-on availability [43, 44]. Such interventions have produced modest success in reducing use and subsequent harms from alcohol and tobacco use [36–41]. Additionally, digitally delivered interventions are likely to be highly acceptable to student populations given the high prevalence of technology use in this population, with almost 90% of young people having access to a smart phone with internet access [45]. However, there has been no review of the effectiveness of online or mobile digital interventions for reducing harm from illicit substance misuse in third-level student populations. With this in mind, we posed the following research question: “Are digital interventions effective in reducing harm from illicit substance misuse in a third-level student population?” This review aims to systematically identify and critically appraise studies examining the effectiveness of digital interventions in reducing harm from illicit substance misuse in a third-level population with a view to examining the overall effectiveness of the interventions.

### Substance misuse

Throughout this review, “substance misuse” is defined as the use of a substance for a purpose not consistent with legal or medical guidelines [22]. For the purpose of this review, the term “substance misuse” will exclude alcohol and tobacco use.

### Harm reduction

The harm reduction approach accepts that a continuing level of substance misuse in society is inevitable, therefore defining its objective as reducing the adverse consequences of substance misuse experienced by the user and others [46]. Harm reduction shifts the focus from measuring substance misuse, to reducing health, social, and economic-related problems [46, 47]. For the purpose of this review, we will include any paper which presents a measure of harm reduction in substance misuse, including but not limited to; a reduction in substance misuse, a reduction in substance misuse-related problems or consequences, or a change in descriptive norms of substance misuse as either a primary or secondary outcome measure.

## Methods

The objective of this review is to collate, summarise and critically appraise the evidence of the effects of digital interventions aiming to reduce harm from substance misuse in third-level students. In accordance with the Preferred Reporting Items for Systematic Reviews and Meta-Analyses (PRISMA) statement, we conducted a systematic review of the published literature, without date restrictions. The search strategy was designed to identify published and unpublished studies in manuscripts, reports and literature available through relevant databases and organisation websites. Academic Search Complete, Cumulative Index to Nursing and Allied Health Literature (CINAHL), MEDLINE, PsycARTICLES, Psychology and Behavioural Sciences Collection, PsycINFO, PubMed, Scopus, Science Direct and the Cochrane databases were searched in April 2018 using the keywords and database specific terms presented in Additional file 1. Search terms focusing on the student population, mHealth intervention type, and substance misuse outcome were used. The keywords were discussed with a librarian prior to commencement. The strategy was also designed to identify grey literature using the search terms “mHealth” AND “substance misuse” AND “student.” We supplemented our electronic search by cross-checking the reference lists of all included studies.

We included studies which published quantitative estimates of the association between the digital intervention and a reduction in harm from substance misuse. Eligibility criteria for inclusion in the systematic review is presented in Table 1.

**Table 1:** Inclusion and Exclusion Criteria

Inclusion Criteria	• Any study deploying a web-based or mobile digital intervention with the aim of reducing harm from substance misuse. • Studies reporting substance misuse as a primary or secondary measure. • Studies reporting a measure of the effectiveness of the intervention. • Studies whose study population consists of students enrolled in third-level institutions (e.g. college or university). • Studies whose definition of “substance misuse” includes any illicit drug, psychoactive drug, or misuse of prescription medication.
Exclusion criteria	• Any study deploying a non-digital intervention only. • Studies reporting a non-third-level population (e.g. young adults, adolescents, secondary school students). • Studies reporting interventions targeting only alcohol and/or tobacco. • Non-English language studies

Initial screening of titles and abstracts were undertaken by two reviewers (SD^1^ and EW) against the inclusion and exclusion criteria. A total of 157 records were identified for screening of title and/or abstract. 126 records were excluded due to irrelevant titles and abstracts. 31 records were included for full text screening. Screening of full text manuscripts for inclusion was undertaken independently by two screeners (SD^1^ and EW). No disagreements on eligibility occurred.

**Fig. 1:**
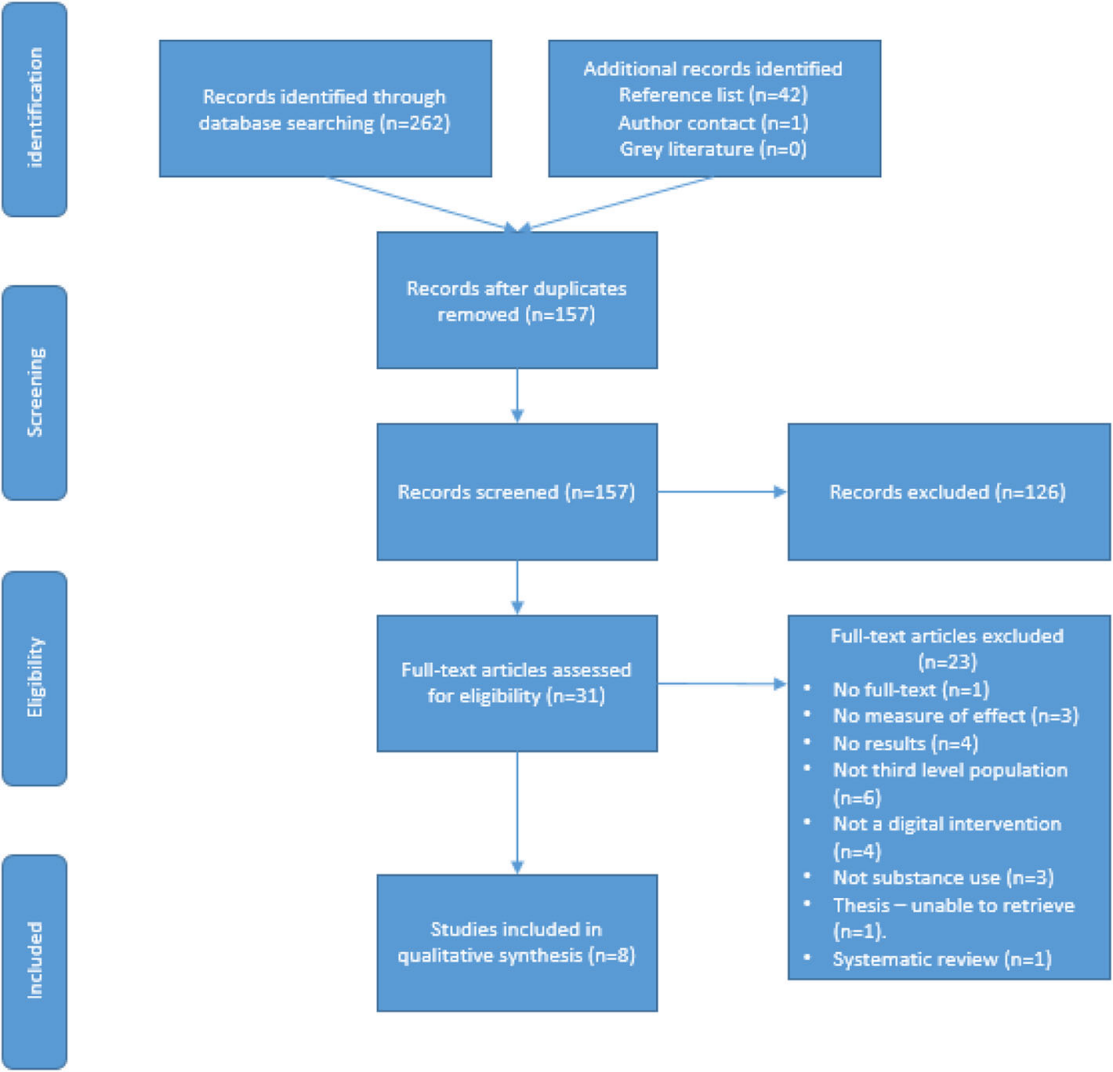
PRISMA flowchart of study selection process

One systematic review was identified during the search strategy which initially met inclusion criteria [42]. The systematic review was appraised using the Joanna Briggs Appraisal Checklist [48]. We found we were unable to produce an update of that systematic review, as the focus was different to ours, dealing with interventions for substances including alcohol and smoking. However, the review did include three papers which dealt with substance misuse, all of which were individually screened for inclusion in this review. One paper [49] did not meet our inclusion criteria, as it examined a computer-based, CD-ROM intervention, as opposed to a web-based, or mobile intervention. The other two papers met our inclusion criteria, and were included in the critical appraisal [50, 51]. The selection process is illustrated in Fig. 1.

### Quality assessment

The quality of included papers was assessed using the Quality Assessment Tool for Quantitative Studies, developed by the Effective Public Health Practice Project [52]. The papers were rated across six domains of: 1. Selection bias 2. Design, 3. Confounders, 4. Blinding, 5. Data collection methods, and 6. Withdrawals and drop-outs. Two reviewers assessed the quality independently (SD^1^ and EW). No disagreements on quality rating occurred. All eligible studies were included in the synthesis irrespective of their assessed quality.

### Data synthesis

Studies were grouped and tabulated according to the variables considered likely to influence the study outcomes and intervention effect. These were; 1. The intervention focus (e.g. whether substance misuse, such as level of misuse, intent to misuse etc., was a primary or secondary measure), 2. The intervention type (e.g. the behaviour-change method employed), 3. The intervention delivery (e.g. delivered online), and 4. The target population (e.g. healthy or substance-using population). Evidence for an intervention effect was considered across studies, in relation to primary and secondary outcomes, and with regard to direction, magnitude, strength and consistency.

### Results

#### Summary of included studies

A total of 8 papers were critically appraised and included in the data synthesis. A brief overview is presented, followed by a summary of the quality rating, strengths and weaknesses, and overall effect measure from each paper. Table 2 summarises the included studies.

**Table 2:** Summary of Included Studies

Author	Study Type	Sample Size	Outcome Measures	Intervention Type	Delivery	Population	Critical Appraisal Score	Overall Result
Lee et al. 2010 [51]	RCT	341	Marijuana use and consequences of use. Also evaluated contemplation to change and family history of drug problems as potential mediators of efficacy	Personalised feedback and descriptive norm correction	Online	Students reporting any use of Marijuana in past 3-months	Moderate	No overall effect on use or consequences. Significant reduction in use in students higher in contemplation.
Elliott & Carey 2012 [50]	CCT	245	Descriptive norms, injunctive norms, marijuana use/initiation	“eTOKE” personalised feedback and descriptive norm correction	Online	Students reporting no marijuana use in past 30 days	Weak	No significant effect on marijuana initiation, but less exaggerated norms.
Elliott et al. 2014 [53]	CCT	317	Marijuana use, problems, user disorder symptoms, and descriptive norms	“eTOKE” personalised feedback and descriptive norm correction	Online	Students reporting past-month marijuana use	Weak	Minimal to small effect for user frequency, problems, disorder symptoms and medium changes for all descriptive norms.
Epton et al. 2014 [54]	RCT	1445	Fruit/veg intake, physical activity, alcohol consumption, smoking, health status, recreational drug use, BMI, health service usage, academic performance, social cognitive variables.	“U@Uni” Self-affirmation manipulation, theory based messages, implementation intention tasks	Online	All incoming undergraduate students	Weak	Increase in recreational drug use in intervention arm
Palfai et al. 2014 [55]	CCT	123	Frequency of marijuana use, marijuana related consequences, readiness to change, perceived norms.	“Marijuana eCHECK UP TO GO” personalised feedback and descriptive norm correction	Online in Student Health Service and off-site	Students attending Student Health Service reporting at least monthly Marijuana use	Moderate	No effect on marijuana use. Medium effect on negative consequences. Significantly reduced student estimates of peer marijuana use.
Cameron et al. 2015 [56]	RCT	2621	Fruit/veg intake, physical activity, alcohol consumption, smoking, health status, recreational drug use, BMI, health service usage, social cognitive variables.	“U@Uni:LifeGuide” Self-affirmation manipulation, theory based messages, implementation intention tasks	Online	All incoming undergraduate students	Weak	Non-significant effect on recreational drug use
Christoff & Boerngen-Lacerda 2015 [57]	CCT	458	ASSIST score (alcohol, tobacco, marijuana, cocaine, amphetamine-stimulants, inhalants, sedatives, hallucinogens, opioids)	“ASSIST/MBI” ASSIST screening and Motivational Brief Intervention	Online on-site	Students with moderate/high ASSIST scores	Weak	Small positive effect, reduction in ASSIST score for marijuana.
Haug et al. 2017 [58]	Pre-Post	1067	Perceived stress, self-management and coping behaviours, interpersonal skills, at risk alcohol use, tobacco smoking, and cannabis use.	“Ready4life” – personalised feedback and weekly messages based on social cognitive theory	Online on-site plus off-site text messages	All students with a mobile phone	Weak	No significant pre-post differences in the percentage of persons using cannabis.

The majority of the studies were presented as Randomised Controlled Trials (RCTs), as well as one pre-post study. Four of the included studies provided inadequate information on the randomisation process so have been labelled a Controlled Clinical Trial (CCT), as per the quality appraisal checklist. Half of the studies examined substance misuse, marijuana in all cases, as their primary outcome. The primary focus of the other studies was on alcohol, smoking, or multiple health behaviours. Sample sizes of the studies ranged from 123 to 2621. Most of the studies were carried out in a university setting. One study was carried out across vocational schools, identified as post-secondary institutions [58]. Three studies recruited the general population of students [54, 56, 58], four studies recruited students reporting current misuse of substances [51, 53, 55, 57], and one study recruited students abstaining from substance misuse [50]. Half of the studies were conducted in the US [50, 51, 53, 55], two were conducted in the United Kingdom (UK) [54, 56], one in Brazil [57] and one in Switzerland [58]. Randomisation was carried out in six studies. The studies could not be combined due to considerable heterogeneity between methodology, outcome measures and statistical analysis.

The included studies assessed harm reduction predominantly in terms of substance misuse or initiation, as consequences or problems, or norm correction. All eight of the studies measured substance misuse, but only two reported a significant reduction in misuse [53, 57]. However, one study also measured contemplation to change in their participants and found that their intervention produced a significant reduction in misuse in students higher in contemplation [51]. Of the four studies measuring substance misuse related harms or consequences, three saw a small [53, 57] to medium [55] reduction in harms or consequences. All three studies measuring changes in peer norms saw less exaggerated norms in the intervention group [50, 53, 55].

The intervention type varied across the studies. The most common intervention type was the personalised feedback and descriptive norm correction, used in four studies [50, 51, 53, 55]. This was designed to prompt self-reflection and consideration of decreased misuse, assessing misuse, pros and cons of misuse, perceived norms, alcohol and cigarette use, related expenses, valued activities, and readiness to change. Participants received feedback on actual norms, annual expenses of substance misuse, health information, campus resources, and tips to decrease misuse. Participation typically took between 20 and 45 min [53]. Three studies used theory-based behaviour techniques, which included self-affirmation theory [54, 56], and social cognitive theory [58]. The self-affirmation tasks aimed to reduce defensive processing of health messages, theory based messages to increase motivation to adopt a healthy lifestyle, and implementation formation to increase the likelihood that good intentions are translated into behaviour [54, 56]. Participants were asked to select their most important personal values and give a reason for their choices. Messages were delivered based on the theory of planned behaviour and were followed by a planner allowing participants to form “if-then” implementation intentions, identifying a good opportunity to act on their intentions, and a suitable response to their identified opportunity. Elements of social cognitive theory including outcome expectations, self-efficacy, observational learning, facilitation and self-regulation were used to develop text messages which focused on substance misuse resistance skills. These were delivered 2–4 times per week over 4 weeks, as part of a 6-month life-skills training programme [58]. One study delivered the Alcohol, Smoking and Substance Involvement Screening Test (ASSIST) questionnaire, followed by a 20 min motivational brief interview using the elements of Feedback, Responsibility, Advice, Menu of Options, Empathy, and Self-Efficacy (FRAMES) [57].

Most of the included studies achieved a weak score on the critical appraisal checklist, with just two achieving moderate quality [51, 55]. Despite seven studies labelling themselves as RCTs, inadequate information on the randomisation process was provided in three studies [50, 53, 57]. A lack of blinding was observed in all of the studies and no study presented evidence of blinding at the data analysis stage. The majority of the studies used self-report outcome measures for substance misuse and related harms and two studies provided no information on the validity of reliability of their outcome measures [50, 53]. However, two studies carried out a biochemical analysis to identify biomarkers of substance misuse in addition to the self-report measures [54, 56]. Three studies suffered from selection bias [54, 56, 57], recruiting less than 60% of the participant sample. However, four studies achieved high completion rates of between 80 and 100% [50, 51, 53, 55]. All study scoring was defined by the Effective Public Health Practice Project quality appraisal tool [52].

Five studies reported at least one positive outcome for harm reduction from substance misuse. Two observed a small reduction in marijuana misuse [53, 57], three reported a reduction in negative consequences of marijuana misuse [53, 55, 57], and three observed a correction in perceived norms around substance misuse [50, 53, 55]. Of these successful interventions, four utilised personalised feedback and norm correction [50, 51, 53, 55] and the other delivered the ASSIST questionnaire followed by Motivational Brief Intervention [57]. The other three studies reported no significant substance misuse harm reduction [54, 56, 58]. The self-affirmation theory saw an increase in recreational substance misuse in the original trial [54], and although this was not repeated in the replication trial, there were no significant effects observed [56]. The social cognitive theory based text messages also saw no significant pre-post differences in the percentage of people using cannabis [58].

## Discussion

This systematic review identified eight studies examining six digital interventions aimed at reducing harm from substance misuse in a third-level student population. Overall, five out of eight studies reported at least one positive outcome for harm reduction from substance misuse in a third level student population. Although study quality was generally weak, these results suggest that this type of intervention may produce some reduction in harm. In terms of safety, one study did produce an increase in substance misuse [54], but substance misuse was not the primary focus of this trial, with substance misuse being measured as one of seven secondary measures. Additionally, this effect was not replicated in a repeat trial of the same intervention. All of the interventions were delivered online, with the majority being delivered from external websites outside of the institutions that participants could access in their own time, from wherever they preferred. However, three of the interventions [55, 57, 58] were delivered (partially) on-site. The online baseline survey for Ready4Life was delivered using tablet computers or mobile phones, within the school classroom followed by text messages sent to the participant’s personal mobile phone over a six-month period [58]. Additionally, the ASSIST/Motivational Brief Intervention was delivered using the researcher’s personal computer. One study [55] examined the impact of on-site vs off-site delivery, with their findings suggesting that on-site web-based interventions may be preferable as students were more likely to complete baseline measures if delivered on-site. However, the use of this method could perhaps limit the capacity of the intervention to reach large numbers of students, thereby reducing the burden on services, which is arguably one of the greatest benefits of interventions of this type. Due to the illegality and stigma associated with substance misuse, participants may be less willing to accurately report their substance misuse while completing an intervention questionnaire on campus, in the presence of researchers or health care professionals.

Unsurprisingly, since marijuana is the most commonly used illicit substance worldwide [59], over half of the studies examined marijuana as the sole substance. Of the studies that reported at least one positive outcome for harm reduction, only one examined multiple substances, but reported an effect only for marijuana misuse [57]. A potential reason for this could be due to participants being more willing to declare marijuana misuse, as it is seen as a less harmful substance [57, 60], and the inconsistent legislation in relation to marijuana misuse between and within countries may result in a higher acceptability of the drug. Recent years have seen substantial declines in the perceived risk of regular marijuana misuse among young adults [1]. However, “harder” substances such as cocaine, ecstasy, heroin etc. still carry a greater stigma associated with misuse, with higher levels of peer disapproval and greater perceived risk [1], perhaps discouraging participants from declaring misuse of these substances.

The overall effectiveness of these interventions may be affected by the place of delivery. Four of the studies were conducted in the US, where there is a between-states variation in the legal status of marijuana. Although the state was not reported in three of the four US studies, from author contact we confirmed the study location. One study was conducted in Boston, Massachusetts [55], which legalised personal use of marijuana in 2016 [61]. Two studies were conducted in New York [50, 53], where marijuana has been decriminalised since the 1970s [62], and the other study was conducted in Washington [51], where personal use was not legalised until 2012 [63]. It is possible that differing legal status, and legislation debates could impact perceptions and norms of use between states. However, only one of the studies [51] acknowledged the potential impact of recent marijuana legalisation debates, or normalisation trends, on the effectiveness of their intervention. Similarly, the differences in legal status of marijuana across countries in this review may limit the generalisability of these results. For example, in the UK, marijuana use, possession, distribution and sale is illegal [64]; in Brazil, marijuana remains illegal, but possession and cultivation for personal use was decriminalised in 2006 [65]; and in Switzerland, marijuana is illegal, but possession of a small amount has been decriminalised since 2013 [66].

Interaction and engagement appeared to be a problem with many of the interventions, Elliott et al. (2014) reported that a “substantial minority” did not remember completing eTOKE and believe this may have contributed to the lack of effect [53]. The Ready4Life program contained 39 activities, but participants only completed on average 15 of these, with only 6.5% completing 36–39 of the activities [58]. Low engagement with the intervention was reported in the U@Uni trial, with only 52% completing the self-affirmation task at 6-month follow-up [54]. However, after changes to the intervention which included reducing the time-consuming baseline questionnaire, solving technical glitches, and using a new platform to create a structured and streamlined process, engagement increased to 85% in the repeat trial [56]. These results suggest that some of the interventions are not engaging enough to have a meaningful and lasting impact on student behaviour. This is a problem commonly seen with this type of intervention, with similar trends reported in online interventions for alcohol [67]. However, the limited face-to-face capacity of health-care professionals remains a barrier to intervention delivery, so despite issues with engagement, digital interventions provide the potential to circumvent this problem [68].

The lack of user-involvement in the design, development and evaluation of such interventions has been a recent focus in the literature [69] suggesting that in order for digital interventions to have the greatest impact, the target user should be involved throughout both the design and evaluation of such interventions. Participatory involvement of the target users through the development process allows for clear articulation of requirements and results in better adherence [70]. Detailed reporting of the design process of such interventions is generally missing from published evaluation articles [69, 71]. Despite the importance of user involvement, only one study presented any detail of user involvement, outlining focus groups used in the pilot testing phase [58]. The User-Centred Design (UCD) process has been identified as integral to the success of digital interventions so it is difficult to interpret the results of an evaluation when these elements have not been described. Although the findings may have internal validity, this does not necessarily predict successful implementation of these interventions in a real-world context [72].

## Conclusion

Despite the optimism surrounding the use of digital interventions to reduce harm from non-illicit substance misuse in students, such as alcohol use and smoking [36–41], the evidence presented on the effects of such interventions for illicit substance misuse is weaker. Digitally delivered interventions for substance misuse are likely to require robust evidence of their effectiveness if they are to be adopted on a widespread basis. Furthermore, it must be noted that as the majority of the existing effectiveness studies provide little to no detail about the design and development process of the intervention, it is difficult to predict whether these interventions would achieve similar results when implemented in a real-world setting. It is essential that core user needs are uncovered in the design of these digital interventions to increase the likelihood of addressing user needs and expectations. This may be achieved through UCD techniques such as persona building, story-telling, and role playing [73]. User engagement and acceptability of an intervention are crucial to its success, indicating that UCD processes may be a method of increasing the effectiveness of digital interventions, and should be considered as integral to the design and evaluation process of these interventions.

Digital interventions have seen successes in other areas of alcohol and tobacco use [36–41], so enthusiasm for their application to substance misuse harm reduction is justified. However, the results demonstrated in this review suggests that the success of interventions for alcohol and tobacco use may not be realised in relation to the misuse of illicit substances, despite the similarities in behaviour change mechanisms and intervention delivery. Previous research focusing on the efficacy of digital interventions to reduce alcohol related harm highlight that these interventions are beneficial, particularly among “groups less likely to access traditional alcohol-related services, such as women, young people, and at-risk users” [67]. However, previous research also highlight the need for future RCTs to ensure efficacy in this population. It would be prudent to consider the differences between alcohol, and illicit substance misuse behaviours before drawing comparisons between the effects of respective interventions. It could be argued that the illegality of substance misuse makes interventions very different to those designed for legal activities such as tobacco smoking and alcohol consumption. Therefore, incorporating the user experience is of even greater importance in the development of such interventions, thus ensuring that each element of the intervention is accessible, engaging and acceptable to the target population.

This study has several strengths and limitations which should be noted. A rigorous search strategy was used, including ten databases, a grey literature search and reference list screening, minimising the potential for missing relevant studies. Additionally, the use of two reviewers throughout the screening and quality appraisal processes minimised the risk of bias. As the included papers in this review are applicable only to third-level students, this may limit the generalisability of the results to a wider population of young adults. Despite the grey literature search, it is possible that unpublished evidence has been missed throughout this review. Similarly, the inclusion of only English-language studies may have excluded some relevant studies.

### Future recommendations

This study conducted a review of the effectiveness of digital interventions for reducing harm from substance misuse in a third-level student population. Unfortunately, the overall quality of the included studies was weak, which means that we cannot definitively draw conclusions regarding the effectiveness of these interventions. However, the moderate successes of digital interventions in both smoking cessation and alcohol harm-reduction support the notion that interventions for substance misuse may have similar impacts. Although not the focus of this study, it has been widely acknowledged that digital interventions are more likely to be successful in populations that have played an active role in their design [70, 72]. None of the studies presented details of the design process so it is difficult to infer the effectiveness of the interventions when used under real-world conditions.

However, as the majority of the interventions did succeed in producing at least one positive harm-reduction measure, and there appeared to be no concerning negative effects overall, we can conclude that these types of intervention do hold promise and more research is required. In particular, future interventions should employ a UCD approach throughout the design, development and evaluation of interventions to elicit the potential of digital interventions for substance misuse harm reduction in a third-level population. Additionally, very few of the studies in this review focused primarily on illicit substances. Interventions solely targeting illicit substances may help to isolate the true effects of the intervention. Of the included studies that did primarily target illicit substances, the focus was only on marijuana. Although marijuana is the most commonly misused substance, many other substances are widely misused [1]. With the changing trends in substance misuse, it is difficult to determine whether similar effects would be seen for other substances. Future interventions should be developed with this in mind, and target a number of commonly misused substances.

## Supplementary information

**Additional file 1.** General search terms. (DOCX 13 kb)

## Data Availability

Not applicable.

## Abbreviations

ASSIST: Alcohol, Smoking and Substance Involvement Screening Test
CCT: Controlled Clinical Trial
CINAHL: Cumulative Index to Nursing and Allied Health Literature
FRAMES: Feedback, Responsibility, Advice, Menu of Options, Empathy, and Self-Efficacy
PRISMA: Preferred Reporting Items for Systematic review and Meta-Analysis
RCT: Randomised Controlled Trial
UCD: User-Centred Design
UK: United Kingdom
US: United States
