# Supplementary material for: A systematic review of the effectiveness of digital interventions for illicit substance misuse harm reduction in third-level students
Source: BMC Public Health. 2019 Sep 9;19:1244. doi: 10.1186/s12889-019-7583-6 (PMC6734361; doi:10.1186/s12889-019-7583-6)
Supplement: Supplementary file 1 — Additional file 1. General search terms. (DOCX 13 kb) [file 12889_2019_7583_MOESM1_ESM.docx]

**Appendix A - General Search Terms**

| **mHealth** | **AND** | **Substance Misuse** | **AND** | **Students** |
| --- | --- | --- | --- | --- |
| mHealth |  | Substance *use |  | College student |
| Mobile health |  | Drug *use |  | University student |
| m-health |  | Prescription drug *use |  | Third-level student |
| Mobile phone intervention |  | Illicit drug *use |  | Post-secondary student |
| Mobile app |  | Illegal drug *use |  | Undergraduate |
| Mobile |  | Recreational drug *use |  | Postgraduate |
| Smart phone app |  | Study drug *use |  | Tertiary student |
| eHealth |  | Smart drug *use |  | Tertiary institute |
| Web-based intervention |  | Psychoactive drug *use |  | University |
| Internet based intervention |  | Problematic drug *use |  | Database Specific Keyword for “students” |
| Internet supported intervention |  | Narcotic *use |  |  |
| Online interventions |  | Cannabis *use |  |  |
| Health information technology intervention |  | Marijuana *use |  |  |
| Social media intervention |  | Database Specific Keyword for “substance use” |  |  |
| Digital Behaviour Change Intervention |  |  |  |  |
| Database Specific Keyword for “mHealth” |  |  |  |  |
